# Supplementary material for: Anemia, micronutrient deficiency, and elevated biomarkers of inflammation among women and children in two districts in the Northern Region of Ghana: A pilot study
Source: PLoS One. 2025 Jun 17;20(6):e0317647. doi: 10.1371/journal.pone.0317647 (PMC12173369; doi:10.1371/journal.pone.0317647)
Supplement: S1 Table — (DOCX) [file pone.0317647.s001.docx]

**S1 Table** Definitions and cut-offs used for the binary outcomes

| Binary outcome | WRA (15-49 y) | PSC (2-5 y) |
| --- | --- | --- |
| Anemia or low Hb [1] |  |  |
| Any anemia | < 120 g/L | < 110 g/L |
| Mild anemia | 110 - 119 g/L | 100 – 109 g/L |
| Moderate anemia | 80 - 109 g/L | 70 - 99 g/L |
| Severe anemia | < 80 g/L | < 70 g/L |
| ID |  |  |
| Low SF [2] | < 15 µg/L | < 12 µg/L |
| Elevated sTfR [3] | > 8.3 mg/L | > 8.3 mg/L |
| Low BIS [4] | < 0 mg/kg | < 0 mg/kg |
| IDA [5] | Hb < 120 g/L and SF < 15 µg/L | Hb < 120 g/L and SF < 12 µg/L |
| Zinc deficiency |  |  |
| Low serum zinc [6] | < 70 µg/dL (fasting) or < 66 µg/dL (non-fasting) | < 65 µg/dL |
| Vitamin A deficiency |  |  |
| Low SR [7] | < 0.70 µmol/L | < 0.70 µmol/L |
| Low serum RBP [8] | < 0.52 µmol/L | < 0.54 µmol/L |
| Vitamin B-12 deficiency |  |  |
| Low serum B-12 [9] | < 221 pmol/L | < 221 pmol/L |
| Folate deficiency |  |  |
| “Insufficient” erythrocyte folate [10, 11] | < 748 nmol/L |  |
| Low erythrocyte folate [11, 12] | < 305 nmol/L |  |
| Low serum folate [11, 12] | < 7 nmol/L |  |
| Elevated AGP | > 1.0 g/L | > 1.0 g/L |
| Elevated CRP | > 5 mg/L | > 5 mg/L |
| Abbreviations: AGP, alpha-1-acid glycoprotein; BIS, body iron stores; CRP, C-Reactive Protein; Hb, Hemoglobin; ID, iron deficiency; IDA, iron deficiency anemia; PSC, Pre-school children; RBP, retinol binding protein; SF, serum ferritin; SR, serum retinol; sTfR, soluble transferrin receptor; WRA, women of reproductive age | | |

**References**

1. WHO. Guideline on haemoglobin cutoffs to define anaemia in individuals and populations. Licence: CC BY-NC-SA 3.0 IGO [Internet]. World Health Organization, Geneva, Switzerland. Available from: <https://iris.who.int/bitstream/handle/10665/376196/9789240088542-eng.pdf?sequence=1>. 2024.

2. WHO. Guideline on use of ferritin concentrations to assess iron status in individuals and populations [Internet]. World Health Organization, Geneva, Switzerland [cited 2024 May 03]. Available from: <https://apps.who.int/iris/handle/10665/331505>,. 2020.

3. Erhardt JG, Estes JE, Pfeiffer CM, Biesalski HK, Craft NE. Combined measurement of ferritin, soluble transferrin receptor, retinol binding protein, and C-reactive protein by an inexpensive, sensitive, and simple sandwich enzyme-linked immunosorbent assay technique. J Nutr. 2004;134(11):3127-32. doi: 10.1093/jn/134.11.3127. PubMed PMID: 15514286.

4. Cook JD, Flowers CH, Skikne BS. The quantitative assessment of body iron. Blood. 2003;101(9):3359-64. Epub 2003/01/11. doi: 10.1182/blood-2002-10-3071. PubMed PMID: 12521995.

5. WHO. Iron deficiency anaemia: assessment, prevention and control: A guide for programme managers. WHO/NHD/01.3 [Internet]. World Health Organization, Geneva, Switzerland [cited 2024 May 04]. Available from: <https://www.who.int/publications/m/item/iron-children-6to23--archived-iron-deficiency-anaemia-assessment-prevention-and-control>. . 2001.

6. Brown KH, Rivera JA, Bhutta Z, Gibson RS, King JC, Lonnerdal B, et al. International Zinc Nutrition Consultative Group (IZiNCG) technical document #1. Assessment of the risk of zinc deficiency in populations and options for its control. Food and nutrition bulletin. 2004;25(1 Suppl 2):S99-203. Epub 2007/12/01. PubMed PMID: 18046856.

7. WHO. Serum retinol concentrations for determining the prevalence of vitamin A deficiency in populations (WHO/NMH/NHD/MNM/11.3) [Internet]. Vitamin and Mineral Nutrition Information System. World Health Organization, Geneva, Switzerland [cited 2024 May 03]. Available from: <http://www.who.int/vmnis/indicators/retinol.pdf>. 2011.

8. Gamble MV, Ramakrishnan R, Palafox NA, Briand K, Berglund L, Blaner WS. Retinol binding protein as a surrogate measure for serum retinol: studies in vitamin A-deficient children from the Republic of the Marshall Islands. The American journal of clinical nutrition. 2001;73(3):594-601. Epub 2001/03/10. doi: 10.1093/ajcn/73.3.594. PubMed PMID: 11237937.

9. Allen LH, Miller JW, de Groot L, Rosenberg IH, Smith AD, Refsum H, et al. Biomarkers of Nutrition for Development (BOND): Vitamin B-12 Review. The Journal of nutrition. 2018;148(suppl_4):1995S-2027S. Epub 2018/12/01. doi: 10.1093/jn/nxy201. PubMed PMID: 30500928; PubMed Central PMCID: PMCPMC6297555.

10. Pfeiffer CM, Sternberg MR, Hamner HC, Crider KS, Lacher DA, Rogers LM, et al. Applying inappropriate cutoffs leads to misinterpretation of folate status in the US population. The American journal of clinical nutrition. 2016;104(6):1607-15. Epub 2016/09/30. doi: 10.3945/ajcn.116.138529. PubMed PMID: 27680995; PubMed Central PMCID: PMCPMC5693380.

11. Pfeiffer CM, Sternberg MR, Zhang M, Fazili Z, Storandt RJ, Crider KS, et al. Folate status in the US population 20 y after the introduction of folic acid fortification. The American journal of clinical nutrition. 2019;110(5):1088-97. Epub 2019/09/11. doi: 10.1093/ajcn/nqz184. PubMed PMID: 31504109; PubMed Central PMCID: PMCPMC6821545.

12. IOM. Dietary reference intakes for thiamin, riboflavin, niacin, vitamin B6, folate, vitamin B12, pantothenic acid, biotin, and choline. Standing Committee on the Scientific Evaluation of Dietary Reference Intakes and its Panel on Folate, Other B Vitamins, and Choline [Internet]. National Academies Press, Washington, D. C., USA [cited 2025 Apr 11]. Available from: <https://www.ncbi.nlm.nih.gov/books/NBK114310/>. 1998
