# Supplementary material for: Anemia, micronutrient deficiency, and elevated biomarkers of inflammation among women and children in two districts in the Northern Region of Ghana: A pilot study
Source: PLoS One. 2025 Jun 17;20(6):e0317647. doi: 10.1371/journal.pone.0317647 (PMC12173369; doi:10.1371/journal.pone.0317647)
Supplement: S2 Table — (DOCX) [file pone.0317647.s002.docx]

**S2 Table** Potential independent variables for bivariate analysis of anemia, micronutrient deficiencies, and elevated biomarkers of inflammation among women of reproductive age and preschool children

| **Potential predictor** | **Variable type** |
| --- | --- |
| **Women of reproductive age (WRA)** |  |
| *Community-level variables* |  |
| Area of residence = “rural” | Nominal (Yes/No) |
| District of residence = Kumbungu | Nominal (Yes/No) |
| *Household-level variables* |  |
| Number of children under 5 y | Discrete |
| Household size | Discrete |
| Household head’s maximum educ. level = Primary | Nominal (Yes/No) |
| Household head’s maximum educ. level = Secondary | Nominal (Yes/No) |
| Household head’s maximum educ. level > Secondary | Nominal (Yes/No) |
| Assets index | Ratio |
| Household Food Insecurity Access Scale (HFIAS) score | Ratio |
| Source of drinking water = Improved | Nominal (Yes/No) |
| Type of toilet facility = Improved | Nominal (Yes/No) |
| Bouillon intake via Adult Male Equivalent (AME) method, g | Ratio |
| Livestock index | Ratio |
| *Individual-level variables* |  |
| Age, y | Ratio |
| Married: Being married | Nominal (Yes/No) |
| Level of formal education = Preschool | Nominal (Yes/No) |
| Level of formal education = Primary | Nominal (Yes/No) |
| Level of formal education = Secondary | Nominal (Yes/No) |
| Employment status = home, informal | Nominal (Yes/No) |
| Employment status = student | Nominal (Yes/No) |
| Typical week’s servings of fruits | Discrete |
| Typical week’s servings of vegetable | Discrete |
| Typical week’s servings of sweets | Discrete |
| Typical week’s servings of salty snack | Discrete |
| Typical week’s servings of sugar sweetened beverages | Discrete |
| Consumed micronutrient supplement in past 30 d | Nominal (Yes/No) |
| Had ≥ 3 loose stools in 24 h in the past 7 days | Nominal (Yes/No) |
| Had fever in the last 7 days | Nominal (Yes/No) |
| Body Mass Index, kg/m^2^ | Ratio |
| Days since last menstrual period start | Ratio |
| Received malaria treatment in the last 4 weeks | Nominal (Yes/No) |
| Received Vitamin A capsules for most recent birth = Yes | Nominal (Yes/No) |
| Received Vitamin A capsules for most recent birth = Don’t know | Nominal (Yes/No) |
|  |  |
| **Preschool children (PSC) 24-59 months** |  |
| Community level variables |  |
| Area of residence = “rural” | Nominal (Yes/No) |
| District of residence = Kumbungu | Nominal (Yes/No) |
| *Household-level variables* |  |
| Household size | Discrete |
| Number of children under 5 yr | Discrete |
| Household head’s maximum educ. level = Primary | Nominal (Yes/No) |
| Household head’s maximum educ. level = Secondary | Nominal (Yes/No) |
| Household head’s maximum educ. level > Secondary | Nominal (Yes/No) |
| Assets index | Ratio |
| HFIAS score | Ratio |
| Source of drinking water = Improved | Nominal (Yes/No) |
| Type of toilet facility = Improved | Nominal (Yes/No) |
| Bouillon intake via AME method, g | Ratio |
| Livestock index | Ratio |
| *Individual-level predictors* |  |
| Age, y | Ratio |
| Child sex = Female | Nominal (Yes/No) |
| Child currently breastfeeding | Nominal (Yes/No) |
| Level in school = Preschool | Nominal (Yes/No) |
| Child’s level in school = Primary | Nominal (Yes/No) |
| Typical week’s servings of fruits | Discrete |
| Typical week’s servings of vegetable | Discrete |
| Typical week’s servings of sweets | Discrete |
| Typical week’s servings of salty snack | Discrete |
| Typical week’s servings of sugar sweetened beverages | Discrete |
| Consumed micronutrient supplement in past 30 d | Nominal (Yes/No) |
| Consumed micronutrient powder in past 30 d | Nominal (Yes/No) |
| Had ≥ 3 loose stools in 24 h in the past 7 days | Nominal (Yes/No) |
| Had fever in the last 7 days | Nominal (Yes/No) |
| Mid-upper arm circumference, cm | Ratio |
| Height-for-age z-score (HAZ) | Ratio |
| Weight-for-height z-score (WHZ) | Ratio |
| Received malaria treatment in the last 4 weeks | Nominal (Yes/No) |
| Received high dose vitamin A in the past 6 months | Nominal (Yes/No) |
