# Supplementary material for: Anemia, micronutrient deficiency, and elevated biomarkers of inflammation among women and children in two districts in the Northern Region of Ghana: A pilot study
Source: PLoS One. 2025 Jun 17;20(6):e0317647. doi: 10.1371/journal.pone.0317647 (PMC12173369; doi:10.1371/journal.pone.0317647)
Supplement: S3 Table — (DOCX) [file pone.0317647.s003.docx]

**S3 Table** Bivariate analysis of potential independent variables for anemia, micronutrient deficiency, and inflammation among women of reproductive age and preschool children^1^

| Target groups and predictor variables | Anemia | | Iron deficiency | | Zinc deficiency | | VA deficiency | | B-12 deficiency | | Folate deficiency | | Inflammation | |
| --- | --- | --- | --- | --- | --- | --- | --- | --- | --- | --- | --- | --- | --- | --- |
|  | Estimate (n) | P | Estimate (n) | P | Estimate (n) | P | Estimate (n) | P | Estimate (n) | P | Estimate (n) | P | Estimate (n) | P |
| **Women of reproductive age** |  |  |  |  |  |  |  |  |  |  |  |  |  |  |
| *Community-level variables* |  |  |  |  |  |  |  |  |  |  |  |  |  |  |
| Area of residence = “rural” | 1.44 (220) | 0.22 | 0.66 (216) | 0.13 | 1.87 (219) | 0.19 | 1.57 (202) | 0.17 | 1.88 (209) | 0.25 | 0.8 (208) | 0.61 | 1.25 (216) | 0.61 |
| District of residence = Kumbungu | 0.94 (220) | 0.84 | 1.36 (216) | 0.27 | 0.47 (219) | 0.13 | 1.03 (202) | 0.92 | 0.77 (209) | 0.63 | 0.55 (208) | 0.19 | 0.83 (216) | 0.66 |
| *Household-level variables* |  |  |  |  |  |  |  |  |  |  |  |  |  |  |
| Household size | 0.96 (219) | 0.12 | 0.99 (215) | 0.61 | 1 (218) | 0.98 | 0.99 (201) | 0.75 | 1.00 (208) | 0.92 | 0.9 (207) | 0.04 | 1.00 (215) | 0.96 |
| Number of children under 5 y | 0.71 (219) | 0.03 | 1.09 (215) | 0.51 | 0.86 (218) | 0.34 | 1.24 (201) | 0.11 | 0.6 (208) | 0.048 | 1.06 (207) | 0.76 | 1.07 (215) | 0.70 |
| Household head’s max. educ. level = Primary | 0.79 (219) | 0.96 | 0.82 (215) | 0.54 | 1.81 (218) | 0.35 | 1.03 (201) | 0.006 | 1.47 (208) | 0.81 | 1.45 (207) | 0.92 | 5.55 (215) | 0.015 |
| Household head’s max. educ. level = Secondary | 1.02 (219) | 0.96 | 1.41 (215) | 0.54 | 2.35 (218) | 0.35 | 1.6 (201) | 0.006 | 0.95 (208) | 0.81 | 1.18 (207) | 0.92 | 2.39 (215) | 0.015 |
| Household head’s max. educ. level > Secondary | 0.99 (219) | 0.96 | 1.48 (215) | 0.54 | 1.38 (218) | 0.35 | 0.12 (201) | 0.006 | 0.6 (208) | 0.81 | 0.85 (207) | 0.92 | 0.65 (215) | 0.015 |
| Assets index | 1.02 (219) | 0.87 | 1.02 (215) | 0.89 | 0.99 (218) | 0.98 | 0.92 (201) | 0.61 | 0.68 (208) | 0.12 | 0.87 (207) | 0.51 | 0.96 (215) | 0.84 |
| HFIAS score | 1.00 (219) | 0.89 | 1.09 (215) | 0.013 | 1.01 (218) | 0.78 | 1.05 (201) | 0.24 | 1.08 (208) | 0.17 | 1.06 (207) | 0.27 | 1.06 (215) | <0.001 |
| Source of drinking water = Improved | 0.44 (219) | 0.032 | 0.46 (215) | 0.03 | 0.66 (218) | 0.41 | 0.43 (201) | 0.05 | 2.83 (208) | 0.13 | 0.74 (207) | 0.58 | 1.52 (215) | 0.40 |
| Type of toilet facility = Improved | 0.83 (219) | 0.60 | 0.58 (215) | 0.09 | 1.58 (218) | 0.31 | 0.78 (201) | 0.52 | 0.38 (208) | 0.12 | 0.95 (207) | 0.92 | 1.05 (215) | 0.92 |
| Bouillon intake via AME method, g | 1.25 (218) | 0.063 | 1.26 (214) | 0.065 | 0.74 (217) | 0.039 | 0.94 (200) | 0.68 | 1.08 (207) | 0.67 | 1.17 (206) | 0.32 | 0.91 (214) | 0.62 |
| Livestock index | 0.82 (219) | 0.30 | 1.05 (215) | 0.75 | 1 (218) | 0.99 | 0.94 (201) | 0.78 | 0.97 (208) | 0.93 | 1.03 (207) | 0.9 | 0.74 (215) | 0.29 |
| *Individual-level predictors* |  |  |  |  |  |  |  |  |  |  |  |  |  |  |
| Age, y | 1.01 (219) | 0.74 | 1.02 (215) | 0.22 | 0.96 (218) | 0.064 | 1.01 (201) | 0.67 | 1.01 (208) | 0.71 | 1.02 (207) | 0.36 | 0.96 (215) | 0.063 |
| Being married | 0.81 (217) | 0.54 | 1.64 (213) | 0.12 | 0.63 (216) | 0.29 | 0.59 (199) | 0.14 | 0.39 (206) | 0.046 | 1.98 (205) | 0.23 | 0.49 (213) | 0.10 |
| Level of formal education = Preschool | 1.52 (218) | 0.43 | 0.41 (214) | 0.74 | 0.63 (217) | 0.65 | 1.53 (200) | 0.40 | 0 (207) | 0.80 | 4.84 (206) | 0.45 | 0 (214) | 0.021 |
| Level of formal education = Primary | 2.12 (218) | 0.43 | 0.8 (214) | 0.74 | 2.02 (217) | 0.65 | 0.33 (200) | 0.40 | 0.72 (207) | 0.80 | 0.65 (206) | 0.45 | 4.27 (214) | 0.021 |
| Level of formal education = Secondary | 1.41 (218) | 0.43 | 0.73 (214) | 0.74 | 0.82 (217) | 0.65 | 1.12 (200) | 0.40 | 1.08 (207) | 0.80 | 0.54 (206) | 0.45 | 3.49 (214) | 0.021 |
| Employment status = home, informal | 1.40 (217) | 0.36 | 0.51 (213) | 0.20 | 0.86 (216) | 0.74 | 0.69 (199) | 0.55 | 1.37 (206) | 0.79 | 0.63 (205) | 0.99 | 0.88 (213) | 0.050 |
| Employment status = student | 1.70 (217) | 0.36 | 0.82 (213) | 0.20 | 1.42 (216) | 0.74 | 1.24 (199) | 0.55 | 0.84 (206) | 0.79 | 1 (205) | 0.99 | 3.44 (213) | 0.050 |
| Typical week’s servings of fruits | 1.05 (217) | 0.49 | 1.11 (213) | 0.16 | 1.08 (216) | 0.44 | 0.97 (199) | 0.74 | 0.86 (206) | 0.27 | 0.95 (205) | 0.68 | 0.98 (213) | 0.86 |
| Typical week’s servings of vegetable | 1.01 (217) | 0.50 | 1.03 (213) | 0.11 | 1.01 (216) | 0.75 | 1.00 (199) | 0.98 | 0.99 (206) | 0.58 | 0.99 (205) | 0.72 | 1.01 (213) | 0.008 |
| Typical week’s servings of sweets | 1.06 (216) | 0.45 | 0.97 (212) | 0.66 | 1.04 (215) | 0.68 | 1.03 (198) | 0.72 | 1.18 (205) | 0.09 | 0.86 (204) | 0.27 | 1.11 (212) | 0.25 |
| Typical week’s servings of salty snack | 1.01 (217) | 0.91 | 1.11 (213) | 0.15 | 1.26 (216) | 0.075 | 1.13 (199) | 0.11 | 0.98 (206) | 0.84 | 1.08 (205) | 0.49 | 1.12 (213) | 0.21 |
| Typical week’s servings of SSBs | 0.98 (217) | 0.78 | 1.06 (213) | 0.30 | 1.04 (216) | 0.55 | 1.18 (199) | 0.005 | 1.06 (206) | 0.49 | 0.99 (205) | 0.9 | 1.04 (213) | 0.57 |
| Consumed micronutrient supplement in past 30 d | 2.08 (213) | 0.14 | 0.99 (209) | 0.99 | 6.42 (212) | 0.10 | 0.55 (196) | 0.37 | 2.12 (202) | 0.26 | 0.36 (201) | 0.33 | 1.09 (209) | 0.90 |
| Had ≥3 loose stools in 24 h in the past 7 d | 2.17 (220) | 0.45 | 3.68 (216) | 0.27 | 0.92 (219) | 0.95 | 0.87 (202) | 0.91 | 0 (209) | 1.00 | 4.32 (208) | 0.23 | 1.00 (216) | 1.00 |
| Had fever in the last 7 d | 1.19 (220) | 0.59 | 0.72 (216) | 0.29 | 2.49 (219) | 0.06 | 0.73 (202) | 0.41 | 1.34 (209) | 0.57 | 0.62 (208) | 0.38 | 1.55 (216) | 0.29 |
| Body Mass Index, kg/m2 | 0.97 (218) | 0.49 | 1.05 (215) | 0.15 | 1.01 (218) | 0.80 | 0.91 (201) | 0.09 | 0.89 (208) | 0.17 | 1.01 (207) | 0.81 | 1.01 (215) | 0.87 |
| Days since last menstrual period start | 0.98 (104) | 0.50 | 0.98 (102) | 0.32 | 1.01 (103) | 0.66 | 0.98 (96) | 0.45 | 0.98 (98) | 0.56 | 0.99 (97) | 0.81 | 0.99 (102) | 0.87 |
| Received malaria treatment in the last 4 weeks | 0.67 (220) | 0.50 | 0.50 (216) | 0.21 | 2.42 (219) | 0.30 | 0.75 (202) | 0.63 | 2.87 (209) | 0.14 | 0.58 (208) | 0.61 | 0.74 (216) | 0.71 |
| Took Vit A capsules for most recent birth = Yes | 1.61 (220) | 0.28 | 1.22 (216) | 0.94 | 0.18 (219) | 0.066 | 0.64 (202) | 0.86 | 5.49 (209) | 0.039 | 0 (208) | 0.22 | 1.82 (216) | 0.47 |
| Took Vit A capsules for most recent birth = DK | 1.68 (220) | 0.28 | 1.08 (216) | 0.94 | 0.49 (219) | 0.066 | 0.97 (202) | 0.86 | 4.23 (209) | 0.039 | 0.72 (208) | 0.22 | 1.71 (216) | 0.47 |
| **Preschool children 2-5 y** |  |  |  |  |  |  |  |  |  |  |  |  |  |  |
| *Community level variables* |  |  |  |  |  |  |  |  |  |  |  |  |  |  |
| Area of residence = “rural” | 0.52 (236) | 0.019 | 0.4 (166) | 0.030 | 2.41 (166) | 0.012 | 0.66 (147) | 0.31 | 4.33 (141) | 0.004 |  |  | 0.84 (166) | 0.59 |
| District of residence = Kumbungu | 1.22 (236) | 0.47 | 1.81 (166) | 0.16 | 0.56 (166) | 0.10 | 3.51 (147) | 0.002 | 1.44 (141) | 0.41 |  |  | 0.94 (166) | 0.86 |
| *Household-level variables* |  |  |  |  |  |  |  |  |  |  |  |  |  |  |
| Household size | 1.04 (234) | 0.11 | 1.02 (164) | 0.60 | 1.03 (164) | 0.40 | 0.97 (145) | 0.41 | 0.98 (139) | 0.62 |  |  | 0.95 (164) | 0.11 |
| Number of children under 5 y | 1.26 (234) | 0.083 | 1.07 (164) | 0.68 | 1.37 (164) | 0.11 | 0.89 (145) | 0.54 | 1.41 (139) | 0.10 |  |  | 0.88 (164) | 0.45 |
| Household head’s max. level = Primary | 0.93 (234) | 0.88 | 2.19 (164) | 0.18 | 1.03 (164) | 0.60 | 0.91 (145) | 0.87 | 1.26 (139) | 0.89 |  |  | 0.62 (164) | 0.16 |
| Household head’s max. educ. level = Secondary | 1.05 (234) | 0.88 | 2.55 (164) | 0.18 | 0.73 (164) | 0.60 | 0.86 (145) | 0.87 | 1.56 (139) | 0.89 |  |  | 0.63 (164) | 0.16 |
| Household head’s max. educ. level > Secondary | 0.76 (234) | 0.88 | 2.70 (164) | 0.18 | 0.51 (164) | 0.60 | 0.58 (145) | 0.87 | 1.35 (139) | 0.89 |  |  | 0.28 (164) | 0.16 |
| Assets index | 0.91 (234) | 0.49 | 1.14 (164) | 0.51 | 0.97 (164) | 0.85 | 0.72 (145) | 0.12 | 1.07 (139) | 0.80 |  |  | 0.89 (164) | 0.49 |
| HFIAS | 0.98 (234) | 0.58 | 1.06 (164) | 0.19 | 0.97 (164) | 0.54 | 1.00 (145) | 1.00 | 1.00 (139) | 0.99 |  |  | 1.00 (164) | 0.93 |
| Source of drinking water = Improved | 1.79 (234) | 0.11 | 1.23 (164) | 0.66 | 0.91 (164) | 0.83 | 2.00 (145) | 0.20 | 1.31 (139) | 0.63 |  |  | 1.60 (164) | 0.27 |
| Type of toilet facility = Improved | 1.31 (234) | 0.40 | 1.02 (164) | 0.96 | 0.39 (164) | 0.020 | 1.41 (145) | 0.48 | 0.58 (139) | 0.39 |  |  | 0.77 (164) | 0.51 |
| Bouillon intake via AME method, g | 1.08 (232) | 0.73 | 0.89 (162) | 0.69 | 1.28 (162) | 0.41 | 1.03 (144) | 0.92 | 1.14 (137) | 0.73 |  |  | 1.43 (162) | 0.18 |
| Livestock index | 1.21 (234) | 0.27 | 0.96 (164) | 0.83 | 1.36 (164) | 0.23 | 1.13 (145) | 0.63 | 1.02 (139) | 0.93 |  |  | 0.96 (164) | 0.83 |
| *Individual-level predictors* |  |  |  |  |  |  |  |  |  |  |  |  |  |  |
| Age, y | 0.97 (232) | 0.033 | 0.98 (163) | 0.17 | 0.97 (163) | 0.075 | 1.01 (144) | 0.70 | 0.97 (138) | 0.27 |  |  | 0.97 (163) | 0.051 |
| Child sex = Female | 0.71 (234) | 0.23 | 0.38 (164) | 0.010 | 0.75 (164) | 0.42 | 0.81 (145) | 0.60 | 0.43 (139) | 0.074 |  |  | 1.14 (164) | 0.69 |
| Child currently breastfeeding | 1.02 (232) | 0.97 | 0.87 (162) | 0.90 | 0.31 (162) | 0.27 | 0 (144) | 0.99 | 0 (138) | 1.00 |  |  | 1.25 (162) | 0.83 |
| Child’s Level in school = Preschool | 1.27 (234) | 0.41 | 1.87 (164) | 0.19 | 0.65 (164) | 0.29 | 1.89 (145) | 0.34 | 0.20 (139) | 0.026 |  |  | 0.61 (164) | 0.39 |
| Child’s level in school = Primary | 0.64 (234) | 0.41 | 2.7 (164) | 0.19 | 0.42 (164) | 0.29 | 0.94 (145) | 0.34 | 0.29 (139) | 0.026 |  |  | 1.17 (164) | 0.39 |
| Typical week’s servings of fruits | 0.75 (232) | 0.006 | 1.04 (162) | 0.74 | 1.10 (162) | 0.37 | 0.75 (144) | 0.04 | 0.92 (138) | 0.55 |  |  | 0.97 (162) | 0.78 |
| Typical week’s servings of vegetable | 0.99 (232) | 0.59 | 1.03 (162) | 0.24 | 0.98 (162) | 0.30 | 1.01 (144) | 0.69 | 1.02 (138) | 0.55 |  |  | 1.02 (162) | 0.41 |
| Typical week’s servings of sweets | 0.92 (232) | 0.14 | 1.07 (162) | 0.35 | 1.00 (162) | 0.96 | 0.91 (144) | 0.32 | 0.96 (138) | 0.63 |  |  | 1.00 (162) | 0.95 |
| Typical week’s servings of salty snack | 0.90 (232) | 0.26 | 1.11 (162) | 0.33 | 1.35 (162) | 0.024 | 1.05 (144) | 0.66 | 0.96 (138) | 0.69 |  |  | 1.00 (162) | 0.98 |
| Typical week’s servings of SSBs | 0.92 (232) | 0.20 | 1.17 (162) | 0.072 | 1.09 (162) | 0.31 | 0.96 (144) | 0.61 | 0.91 (138) | 0.44 |  |  | 0.97 (162) | 0.67 |
| Consumed micronutrient supplement in past 30 d | 1.31 (231) | 0.59 | 0.85 (162) | 0.77 | 0.83 (162) | 0.74 | 0.13 (144) | 0.054 | 1.32 (138) | 0.72 |  |  | 1.05 (162) | 0.93 |
| Consumed micronutrient powder in past 30 d | 1.00 (232) | 1.00 | 0.37 (163) | 0.48 | 0.77 (163) | 0.84 | 0 (145) | 1.00 | 0 (139) | 1.00 |  |  | 0 (163) | 1.00 |
| Had ≥3 loose stools in 24 h in the past 7 d | 0.49 (235) | 0.26 | 0.31 (165) | 0.14 | 2.89 (165) | 0.21 | 3.46 (146) | 0.10 | 1.94 (140) | 0.48 |  |  | 2.16 (165) | 0.28 |
| Had fever in the last 7 d | 1.03 (235) | 0.92 | 0.55 (165) | 0.12 | 1.18 (165) | 0.66 | 2.95 (146) | 0.015 | 1.03 (140) | 0.95 |  |  | 2.7 (165) | 0.01 |
| Mid-upper arm circumference, cm | 0.82 (236) | 0.15 | 1.21 (166) | 0.31 | 0.88 (166) | 0.48 | 0.94 (147) | 0.75 | 0.65 (141) | 0.10 |  |  | 0.83 (166) | 0.28 |
| Height-for-age z-score (HAZ) | 0.76 (227) | 0.008 | 1.08 (160) | 0.59 | 0.92 (160) | 0.50 | 0.89 (141) | 0.46 | 0.57 (137) | 0.009 |  |  | 0.81 (160) | 0.10 |
| Weight-for-height z-score (WHZ) | 1.02 (227) | 0.87 | 1.06 (160) | 0.80 | 1.22 (160) | 0.36 | 1.05 (141) | 0.83 | 0.72 (137) | 0.22 |  |  | 1.10 (160) | 0.64 |
| Received malaria treatment in the last 4 wk | 0.94 (235) | 0.90 | 0.70 (165) | 0.56 | 1.04 (165) | 0.96 | 0.59 (146) | 0.47 | 0.54 (140) | 0.46 |  |  | 3.70 (165) | 0.027 |
| Received high dose vitamin A in the past 6 mo | 1.02 (195) | 0.96 | 0.75 (137) | 0.53 | 0.65 (137) | 0.34 | 0.7 (123) | 0.50 | 0.69 (117) | 0.53 |  |  | 0.58 (137) | 0.24 |

Abbreviations: AME, Adult Male Equivalent; DK, Don’t know; HFIAS, Household Food Insecurity Access Scale; MNP, micronutrient powder; SSB, sugar sweetened beverages; WRA, women of reproductive age

^1^Estimates are odds ratios derived from bivariate logistic regression models.
