# Supplementary material for: Anemia, micronutrient deficiency, and elevated biomarkers of inflammation among women and children in two districts in the Northern Region of Ghana: A pilot study
Source: PLoS One. 2025 Jun 17;20(6):e0317647. doi: 10.1371/journal.pone.0317647 (PMC12173369; doi:10.1371/journal.pone.0317647)
Supplement: Attachment — Submitted filename: 1 Response to Reviewers_22Apr25.docx [file pone.0317647.s004.docx]

**Responses to Reviewers**

**PONE-D-24-60529: Anemia, micronutrient deficiency, and elevated biomarkers of inflammation among women and children in two districts in the Northern Region of Ghana: A pilot study**

**Reviewer #1:**

Thank you for the opportunity to review this manuscript.
This manuscript is a valuable contribution to understanding anemia and micronutrient deficiencies in Northern Ghana. The manuscript exhibits undeniable strengths, including the robust application of generalized linear mixed models to account for clustering and comprehensive reporting of results. The authors effectively connect their findings to broader public health implications, such as fortification interventions.

Specific areas for improvement:
**I. Major:**

**Comment:**
o Address how missing venous blood samples for 29% of preschool children were handled. Were these participants excluded from analyses, or were imputation techniques applied?

**Response:**

Thanks to the reviewer for this comment. Participants without venous blood samples were excluded from analyses involving biomarkers that required serum samples. The study sample was not designed to be representative of the general population, and so no imputation method was applied. Additionally, even after incorporating the available data in imputation procedures, we would not expect the conditional missingness to be at random. We also note that the challenges in obtaining blood samples were associated with younger children and in analysis, child age was associated with less anemia but not associated with micronutrient deficiencies. Thus, in theory, there may be some error underestimating anemia prevalence among children but it is unlikely our other conclusions would be substantively affected.

We have included the following in **Lines 329-32** of the revised manuscript:

“Participants without venous blood samples were excluded from analyses involving biomarkers that required serum samples. No imputation method was applied for missing biomarker data because the missingness was not at random but was primarily due to technical challenges in obtaining venous samples.”

**Comment:**
o It is unclear if all raw data (e.g., anonymized participant-level data) are included or easily accessible. The phrase “data will be made available three years after data collection” does not comply with the requirement for immediate availability upon publication. Ensure immediate public access to all data, or explicitly justify any restrictions and provide alternative means for researchers to access restricted data.

**Response:**

We have revised the text to read:

“Consistent with policy of the Bill & Melinda Gates Foundation, all de-identified data have been

made available online at  <https://osf.io/t3zrn/files/osfstorage>.”

**II. Minor:**

**Comment:**
o While the study compares findings to the 2017 Ghana Micronutrient Survey, it lacks statistical comparisons to determine the significance of differences.

**Response:**

We agree that statistical comparisons can be useful when comparing prevalence estimates across studies. We did not conduct formal tests of significance for differences between our findings and those from the 2017 Ghana Micronutrient Survey due to obvious study differences, including design and sampling strategies, which limit comparability.

We have included the following in **Lines** **504–7** of the revised manuscript:

“While we did not statistically compare our estimates with those from the 2017 GMS due to obvious differences such as survey design and sampling frames, such descriptive comparisons still offer useful context for interpreting the prevalence patterns observed in our study.”

**Comment:**
o Address potential biases introduced by this approach, particularly in densely populated or inaccessible areas.

**Response:**

Our study was a pilot study in two districts in the Northern Region and was not directly comparable to the 2017 Ghana Micronutrient Survey, which was a national survey. In the initial (**Line 458-459**) and revised (**Line 475-6**) submissions, we acknowledged that excluding the inaccessible part of the Tolon district due to flooding was a weakness of the study. However, we noted in the initial (**Line 460-465**) and revised (**Line 477-86**) submissions that these situations were unlikely to substantially limit the generalizability of our results, since districts were generally homogenous in terms of geographical characteristics, economic status, and dietary practices [1, 2], and the flooding that made some areas in the Tolon district difficult to access was short-lived.

**Comment:**
o Clusters in the Tolon district were chosen based on accessibility. Discuss how this may limit generalizability and how these biases were mitigated.

**Response:**

As noted in the Discussion section (**Lines 475–86**), we acknowledged the potential for selection bias due to excluding inaccessible parts of the Tolon district. However, we noted that this was unlikely to limit the generalizability of our findings across the study districts because these areas are generally homogeneous in terms of geographical, socioeconomic, and dietary characteristics. Furthermore, associations between living in rural community and micronutrient deficiency were only statistically significant for some outcomes (iron deficiency among WRA and PSC, anemia among PSC only, and vitamin B-12 deficiency among PSC only), which suggested that the exclusion of some rural clusters in the Tolon district may have introduced limited bias, however, any such bias is likely to be small given the overall geographic and socioeconomic homogeneity of the district.

We added the following to the Discussion section (**Line 481-6**):

“Given that the associations between living in a rural community and micronutrient deficiency were only significant for some outcomes (i.e., iron deficiency in WRA and PSD, anemia in PSC only, and vitamin B-12 deficiency in PSC only), the exclusion of some rural clusters in the Tolon district may have introduced limited bias; however, any such bias is likely to be small given the overall geographic and socioeconomic homogeneity of the district.”

**Comment:**
o Expand on implications for policy and practice. For example, how can findings inform national micronutrient fortification programs?

**Response:**

Thanks to reviewer for this suggestion. We have expanded the relevant part of Conclusions section as follows (**Lines 578-90**), including how our findings may inform national micronutrient fortification strategies:

“Our findings suggest that current national fortification programs targeting edible oil and wheat flour may not sufficiently reach or impact all subpopulations, particularly in rural or underserved areas. While the use of multiple micronutrient powder in home-fortification [3] might contribute to improving micronutrient intakes of PSC in this setting, its long-term sustainability remains uncertain [4]. Thus, national stakeholders should consider reviewing and potentially expanding the scope of existing fortification efforts. In particular, bouillon fortification has been proposed as a possible strategy to increase dietary micronutrient adequacy among individuals at risk of micronutrient deficiency [5, 6]. Bouillon cubes are widely consumed in Ghana [7] and may serve as an effective additional vehicle for delivering key micronutrients and improving coverage. In our previous analysis [8], 99% of 369 surveyed households reported ever using bouillon in cooking, and 77% reported typically using it two or more times per day. Our data can guide which micronutrients are needed, and help identify the population groups that may benefit most.”

**Comment:**
o Compare results to international data for broader relevance.

**Response:**

We have inserted a brief section (**Lines 513-5**) as follows:

“For instance, the anemia prevalence rates observed in our study (31% in WRA; 36% in PSC) align with the global estimates of 30% among WRA and 40% among PSC [9].”

**Comment:**
o Emphasize actionable recommendations in the conclusion, such as advocating for specific interventions like water sanitation or fortified foods.

**Response:**

We added the conclusion (**Lines 593-8**) to the Conclusions section:

“To reduce micronutrient deficiencies in this and similar settings, potential strategies may include introducing additional delivery vehicles, such as multiple micronutrient-fortified bouillon cubes. Given the observed associations between improved drinking water and reduced risk of anemia and micronutrient deficiencies, ensuring access to safe drinking water may provide additional benefits. Policymakers and program implementers should consider the potential benefits and costs of these multi-sectoral approaches when designing interventions.”

**Reviewer #2: Dear Author
Comments:**
It is a well-planned study and the number of regions-cases evaluated is sufficient. The aims of the study are emphasized in detail. The results are reported in accordance with the aims. The findings are as expected. However, the results of the study will also be a guide for low-income countries. Anemia is a global problem in childhood and lactation group. It has emphasized the problems of micronutrient deficiency and anemia in this group with its results and has constructive suggestions in line with the results. Current results may guide larger studies.
Best regards

**Response**

We thank the reviewer for these comments

**Reviewer #3: Paper Accepted**
And anyone who has conducted human research knows what enormous effort was made to gain permission to participate, as well as collect anthropometric measurements and biological samples.

**Minor Revisions**
**Comments**

Include limitations of Food Frequency Questionnaire (FFQ) to predict actual nutrient intake. A number of studies have shown r-values at or below 0.3 for predicting actual nutrient intake. Over the years, serving sizes of particular foods (for example fruits and vegetables) have varied. A table with serving sizes shown could be added to the paper's supplemental information. Additionally, in this study, this tool limited its questions to include fruits, vegetables, salty snacks, sweetened snacks, and sugar-sweetened beverages and did not appear to include protein sources. Based on the high level of zinc inadequacy, providing a supplemental list of protein food sources to the questionnaire would seem reasonable.

**Response**

Thanks to the Reviewer for these comments. We adapted our FFQ from the World Health Organization STEPS (STEPwise approach to non-communicable disease risk factor surveillance) instrument [10]. We have revised the dietary data collection section (Line **214-24**), including adding how women or caregivers were assisted in estimating intakes of the items:

“Additional individual-level information included participants’ age, education, consumption patterns (typical week’s servings) of selected food groups, use of micronutrient-containing supplements and micronutrient powders in the past 30 d, and morbidity in the past 7 d. The food groups (by a food frequency questionnaire, FFQ) included fruits, vegetables, sweetened snacks (e.g., biscuits, candies or chocolates), salty snacks (e.g., crisps and salty crackers), and sugar-sweetened beverages (SSBs, e.g., soft drinks and sugar-sweetened fruit drinks). The FFQ was adapted from the World Health Organization STEPS (STEPwise approach to non-communicable disease risk factor surveillance) instrument [10]. Showcards with photos of local food items and typical servings sizes were used as a prompt during interviews. A serving of **fruits** or **vegetables** was defined as 80 g [10]. For **SSBs,** a serving was defined as 120 mL, and for **salty snacks** and **sweetened snacks**, each serving was defined as 20 g [11].”

Protein food sources were not included in the STEPS instrument. In the subsequent RCT [12], we used the new IYCF indicators [13], which include animal products, but these were not available at the time of the pilot study, which is why we used STEPS as a starting point.

**Comment:**

Because one of the goals of the study is to determine the potential to use bouillon cubes for micronutrient distribution, it would have been helpful to see what percent of the surveyed households presently use bouillon cubes or were willing to incorporate this into their diet regularly.

**Response**

As indicated in **Line 209-13**, we determined household bouillon consumption over the prior 30 days by using a household questionnaire [14], and estimated participants’ bouillon “apparent intake” through the Adult Male Equivalent method [15].

In addition, we have included the following finding from our previous analysis in **Line 587-9**:

“In our previous analysis [8], 99% of 369 surveyed households reported ever using bouillon in cooking, and 77% reported typically using it two or more times per day.”

**Comment**

Location of raw data should be indicated in manuscript.

**Response**

As indicated above, all de-identified data and forms will be made available online at <https://osf.io/t3zrn/> upon publication of the manuscript.

**Comment**
1) Is the manuscript technically sound, and do the data support the conclusions?
A research study is only as good as its methods. The methods described in this study provide a thorough guide to what was done making it possible to repeat the study. This is a rigorous, with appropriate controls, replication, and sample sizes. Conclusions drawn on anemia and MND are appropriately based on the data presented.

**Response**

We thank the Reviewer for this.

**Comment**

This study's methods are generally accepted as valid. The food frequency questionnaire (FFQ) is often used to estimate the nutritional adequacy of the diet. Sadly, this often used tool has a relatively low correlation coefficient when compared to a 24-hour recall and can lead to misinterpretations. A number of studies have shown r-values at or below 0.3 for predicting actual nutrient intake.

**Response**

We agree with the Reviewer. However, as described above, we believe that our use of showcards with photos of local food items and typical serving sizes (including estimated weights (g) or volumes (mL)) may have improved the accuracy and consistency of participant or caregiver reporting. While we agree that FFQs have limitations for estimating nutrient intake, our focus was on describing consumption patterns of selected food groups rather than on estimating nutritional adequacy.

We have inserted the following in **Line 214-5**:

“Additional individual-level information included participants’ age, education, consumption patterns (typical week’s servings) of selected food groups...”

**Comment**
2) Has the statistical analysis been performed appropriately and rigorously?
Based on data presented, it would appear that statistical data is both appropriate and rigorous.

**Response**

We thank the Reviewer for this.

**Comment**
3) Have the authors made all data underlying the findings in their manuscript fully available?
The supplemental data appears to contain more analyses rather than raw data. Location of raw data should be indicated in manuscript.

**Response**

As indicated above, all de-identified data and forms will be made available online at <https://osf.io/t3zrn/> upon publication of the manuscript.

**Comment**
4) Is the manuscript presented in an intelligible fashion and written in standard English?
Manuscript is well written.

**Response**

We thank the Reviewer for this.

**Comment**
5) As a pilot study, the paper seems to be the first paper of a 2-part study. Follow-up study listed is reference **#27**.

**Response**

This paper reports the results of our pilot study conducted before the RCT mentioned in reference **#16** (previously #27).

**Comment**

Benefits to Science:
See comments above regarding methods.
It was refreshing to see the full complement of micronutrients chosen to evaluate levels of anemia and iron deficiency, as well as inflammation indices. The use of well-established labs to analyze the samples also provides a level of confidence in appropriate laboratory technique.

**Response**

We thank the Reviewer for this.

**Comment**
Discussion
The discussion is the weakest part of the paper. The confusing MND information regarding relationship between FFQ and MND may well be due to the low correlation coefficient when compared to a 24-hour recall, this limitation should be included.

**Response**

We understand the Reviewer’s concern about the use of FFQs. However, as explained above, we assessed the intakes of fruits, vegetables, sweetened snacks, salty snacks, and sugar-sweetened beverages using questions taken from the WHO STEPS instrument [10], which has been widely used for monitoring various outcomes including unhealthy diet [16, 17]. Our reliance on self-report for dietary data collection may have introduced some bias; however, the use of showcards with photos of local food items and typical serving sizes likely improved reporting accuracy and consistency. While we agree that FFQs have limitations for estimating nutrient intake, our focus was on describing consumption patterns of selected food groups rather than on estimating total nutrient intake. We have clarified in the revised manuscript (**Lines 489–92**) that a key limitation is our inability to determine whether the observed associations with micronutrient deficiency reflect a biological mechanism or whether food consumption patterns act as proxies for other unmeasured factors.

In the revised manuscript, we have included the following in **Lines 487-92**:

“Our reliance on self-report for dietary data collection may have introduced some bias; however, the use of showcards with photos of local food items and typical serving sizes likely improved reporting accuracy and consistency. While the FFQ focused on describing consumption patterns of selected food groups, the primary limitation is that we cannot determine whether the associations observed with MNDs reflect underlying biological mechanisms or whether the dietary patterns serve as markers for other unmeasured factors.”

**Comment**

Too often sugary and salty foods are thought to be the cause of malnutrition. My 20 years of clinical observations are that these food choices are instead the result of the nutrient inadequacies rather than the cause.

**Response**

We thank the Reviewer for sharing this observation. We agree that dietary choices may, in some cases, reflect underlying physiological needs or nutrient deficiencies [18, 19], and that reverse causality could be a plausible explanation, particularly in cross-sectional studies such as ours.

We have revised **Lines 541-4** as follows:

“High consumption of SSBs may be linked to lower intake of essential nutrients, as these beverages often provide calories with minimal nutritional value, reducing the consumption of more nutritious options [20]. It is possible there are other explanations for the observed associations [18, 19].”

**Comment**

Also, it might have been helpful to have water samples analyzed for micronutrients and heavy metals that could interfere with iron absorption.

**Response**

We agree that analyzing water and food samples for micronutrients and heavy metals could be informative, particularly given the potential of calcium [21] and cadmium [22], for example, to interfere with iron absorption and increase the risk of anemia. However, this was beyond the scope of the present study.

We have added this consideration to the Discussion for possible future research (**Line 523-6**):

“We did not analyze water and food samples for micronutrients and heavy metals, as some of these metals, including calcium [21] and cadmium [22], could potentially influence iron absorption and anemia risk. Future studies may benefit from including these assessments.”

**Comment**

Bottom-line
Even with the paper’s limitations, the well detailed methodology is of great benefit for those planning to do human research.

**Response**

We thank the Reviewer for this.

**Reviewer #4:**

Researchers have found that anemia and micronutrient deficiencies are common in the Tolon and Kumbungu districts. This study looked again at these issues in lactating women (LW), women of reproductive age (WRA), and preschool children (PSC) to help test fortified bouillon cubes. They wanted to identify what factors contribute to anemia, micronutrient deficiencies, and inflammation among WRA and PSC. They also examined how often anemia occurs along with these other issues. Even though Ghana has programs to improve micronutrient intake, many WRA and PSC in these districts still face high rates of deficiencies. The researchers suggest that more strategies are needed to reduce these deficiencies in these areas and similar ones.

This is a study with valuable results. All sections of the article are well-written. Below are several comments for your consideration:

**Comment 1:**
In the introduction, please define micronutrients and explain which factors are considered to be micronutrients. Additionally, clarify why you selected certain micronutrients over others for this study.

**Response**

In **Line 112**, we have provided “(vitamin and mineral” in parentheses to define micronutrients as [23].

In **Line 142-5**, we have clarified the rationale for selecting specific micronutrients for the present analysis as follows:

“The present analysis focused on the micronutrients including vitamin A, vitamin B-12, folate, iodine, iron, and zinc, which were likely to be of public health importance [24] in Ghana and were intended for inclusion in the micronutrient-fortified bouillon cubes [12].”

**Comment 2:**
At the end of the introduction, is the following sentence accurate?
(Whether the co-occurrence of anemia with MNDs or inflammation significantly contributed to anemia prevalence.)

**Response**

We have revised the study objective in question to read (**Line 149-50**):

“(c) evaluate whether anemia co-occurred with MNDs or inflammation significantly more frequently than expected by chance.”

**Comment 3:**
Discussion section, line 483: The high prevalence of anemia and MNDs among WRA and PSC in this study is consistent with data from other low- and middle-income countries. Besides addressing low- and middle-income countries, compare your results with those of similar studies conducted in high-income countries.

**Response**

We have included the following in **Line 515-9**:

“These rates are relatively high compared with those reported for high-income countries, such as the United States, where anemia prevalence was estimated at 11.8% among WRA and 6.1% among PSC in 2019 [25], while the prevalence of MNDs, including folate (0%), iron (22%), zinc (14%) among WRA in 2022 [26] were considerably lower.”

**References**

1.	GSS. Ghana Living Standards Survey (GLSS) Main Report [Internet]. Ghana Statistical Service, Accra, Ghana [cited 2023 Dec 15]. Avialable from: <https://www.statsghana.gov.gh/gssmain/fileUpload/pressrelease/GLSS7%20MAIN%20REPORT_FINAL.pdf>. 2019.

2.	Marchetta F. On the move livelihood strategies in Northern Ghana. ⟨halshs-00591137⟩. DOI: <https://shs.hal.science/halshs-00591137/>. 2011.

3.	Kyei-Arthur F, Situma R, Aballo J, Mahama AB, Selenje L, Amoaful E, et al. Lessons learned from implementing the pilot Micronutrient Powder Initiative in four districts in Ghana. BMC nutrition. 2020;6(1):50. Epub 2020/12/10. doi: 10.1186/s40795-020-00382-3. PubMed PMID: 33292686; PubMed Central PMCID: PMCPMC7650146.

4.	Zlotkin S. The Challenges of Sustaining Multiple Micronutrient Interventions. Sight and Life. 2021;35(1):71-3.

5.	Adams KP, Vosti SA, Becher E, Ishaya F, Engle-Stone R. Bouillon fortification as a strategy to address inequities in micronutrient adequacy of diets in Nigeria. Annals of the New York Academy of Sciences. 2024;1540(1):235-50. Epub 2024/09/10. doi: 10.1111/nyas.15207. PubMed PMID: 39255239.

6.	Adams KP, Vosti SA, Some JW, Tarini A, Becher E, Koudougou K, et al. Micronutrient-fortified bouillon as a strategy to improve the micronutrient adequacy of diets in Burkina Faso. Annals of the New York Academy of Sciences. 2024;1536(1):135-50. Epub 2024/05/29. doi: 10.1111/nyas.15155. PubMed PMID: 38809659.

7.	GHS/UNICEF/GAIN. National Iodine Survey Report, Ghana 2015 [Internet]. Ghana Health Service, Accra, Ghana [cited 2025 Mar 22]. Available from: <https://www.unicef.org/ghana/media/1296/file/UN735926.pdf>. 2017.

8.	Kumordzie S, Davis JN, Adams KP, Tan X, Adu-Afarwuah S, Wessells KR, et al. Understanding patterns and drivers of bouillon use in Northern Ghana to inform fortification planning. 2021;5:655.

9.	WHO. Anemia [Internet]. World Health Organization, Geneva, Switzerland [cited 2024 Sep 11]. Available from: <https://www.who.int/news-room/fact-sheets/detail/anaemia>. 2023.

10.	WHO. The WHO STEPwise approach to noncommunicable disease risk factor surveillance [Internet]. World Health Organization, Geneva, Switzerland [cited 2024 Nov 8]. Available from: <https://www.who.int/teams/noncommunicable-diseases/surveillance/systems-tools/steps>. 2017.

11.	FDA. Reference Amounts Customarily Consumed: List of Products for Each Product Category: Guidance for Industry. [Internet]. United States Food and Drug Administration., Washington, D.C. [cited 2025 Mar 21]. Available from: <http://www.fda.gov/FoodGuidances>. 2018.

12.	Engle-Stone R, Wessells KR, Haskell MJ, Kumordzie SM, Arnold CD, Davis JN, et al. Effect of multiple micronutrient-fortified bouillon on micronutrient status among women and children in the Northern Region of Ghana: Protocol for the Condiment Micronutrient Innovation Trial (CoMIT), a community-based randomized controlled trial. PloS one. 2024;19(5):e0302968. Epub 2024/05/06. doi: 10.1371/journal.pone.0302968. PubMed PMID: 38709803; PubMed Central PMCID: PMCPMC11073681.

13.	WHO & UNICEF. Indicators for assessing infant and young child feeding practices: definitions and measurement methods. Licence: CC BY-NC-SA 3.0 IGO [Internet]. World Health Organization and the United Nations Children’s Fund, Geneva, Switzerland [cited 2025 Apri 10]. Available from: <https://www.who.int/publications/i/item/9789240018389>. 2021.

14.	Friesen VM, Mbuya MN, Neufeld LMJGAfING, Switzerland. The Fortification Assessment Coverage Toolkit (FACT). 2019.

15.	Weisell R, Dop MC. The adult male equivalent concept and its application to Household Consumption and Expenditures Surveys (HCES). Food and nutrition bulletin. 2012;33(3 Suppl):S157-62. Epub 2012/12/01. doi: 10.1177/15648265120333S203. PubMed PMID: 23193766.

16.	Guthold R, Louazani SA, Riley LM, Cowan MJ, Bovet P, Damasceno A, et al. Physical activity in 22 African countries: results from the World Health Organization STEPwise approach to chronic disease risk factor surveillance. Am J Prev Med. 2011;41(1):52-60. Epub 2011/06/15. doi: 10.1016/j.amepre.2011.03.008. PubMed PMID: 21665063.

17.	Riley L, Guthold R, Cowan M, Savin S, Bhatti L, Armstrong T, et al. The World Health Organization STEPwise Approach to Noncommunicable Disease Risk-Factor Surveillance: Methods, Challenges, and Opportunities. Am J Public Health. 2016;106(1):74-8. Epub 2015/12/24. doi: 10.2105/AJPH.2015.302962. PubMed PMID: 26696288; PubMed Central PMCID: PMCPMC4695948.

18.	Ioannidis JPA. The Challenge of Reforming Nutritional Epidemiologic Research. JAMA. 2018;320(10):969-70. Epub 2018/11/14. doi: 10.1001/jama.2018.11025. PubMed PMID: 30422271.

19.	Gougeon L, Payette H, Morais JA, Gaudreau P, Shatenstein B, Gray-Donald K. A prospective evaluation of the depression-nutrient intake reverse causality hypothesis in a cohort of community-dwelling older Canadians. The British journal of nutrition. 2017;117(7):1032-41. Epub 2017/05/04. doi: 10.1017/S0007114517000782. PubMed PMID: 28462727.

20.	Abubakar H, A., Shahril MR, Mat S. Nutritional status and dietary intake among Nigerian adolescent: a systematic review. BMC Public Health. 2024;24(1):1764.

21.	Lonnerdal B. Calcium and iron absorption--mechanisms and public health relevance. Int J Vitam Nutr Res. 2010;80(4-5):293-9. Epub 2011/04/05. doi: 10.1024/0300-9831/a000036. PubMed PMID: 21462112.

22.	Rawee P, Kremer D, Nolte IM, Leuvenink HGD, Touw DJ, De Borst MH, et al. Iron Deficiency and Nephrotoxic Heavy Metals: A Dangerous Interplay? Int J Mol Sci. 2023;24(6). Epub 2023/03/30. doi: 10.3390/ijms24065315. PubMed PMID: 36982393; PubMed Central PMCID: PMCPMC10049453.

23.	Carnegie RE, Zheng J, Borges MC, Jones HJ, Wade KH, Sallis HM, et al. Micronutrients and Major Depression: A Mendelian Randomisation Study. Nutrients. 2024;16(21). Epub 2024/11/13. doi: 10.3390/nu16213690. PubMed PMID: 39519523; PubMed Central PMCID: PMCPMC11547740.

24.	Darnton-Hill I, Webb P, Harvey PW, Hunt JM, Dalmiya N, Chopra M, et al. Micronutrient deficiencies and gender: social and economic costs. The American journal of clinical nutrition. 2005;81(5):1198S-205S. Epub 2005/05/11. doi: 10.1093/ajcn/81.5.1198. PubMed PMID: 15883452.

25.	WHO. WHO Global Anaemia estimates, 2021 Edition [Internet]. World Health Organization, Geneva, Switzerland [cited 2025 Mar 31]. Available from: <https://www.who.int/data/gho/data/themes/topics/anaemia_in_women_and_children?utm_source=chatgpt.com> 2021.

26.	Stevens GA, Beal T, Mbuya MNN, Luo H, Neufeld LM, Global Micronutrient Deficiencies Research G. Micronutrient deficiencies among preschool-aged children and women of reproductive age worldwide: a pooled analysis of individual-level data from population-representative surveys. The Lancet Global health. 2022;10(11):e1590-e9. Epub 2022/10/15. doi: 10.1016/S2214-109X(22)00367-9. PubMed PMID: 36240826; PubMed Central PMCID: PMCPMC10918648.
